# Anatomical Study of the Application of a Galeo-Pericranial Flap in Oral Cavity Defects Reconstruction

**DOI:** 10.3390/jcm12247533

**Published:** 2023-12-06

**Authors:** Alice Marzi Manfroni, Maria Vittoria Marvi, Simone Lodi, Cyril Breque, Giulio Vara, Alessandra Ruggeri, Giovanni Badiali, Lucia Manzoli, Achille Tarsitano, Stefano Ratti

**Affiliations:** 1 Oral and Maxillo-Facial Surgery Unit, IRCCS Azienda Ospedaliero-Universitaria di Bologna, Via Albertoni 15, 40138 Bologna, Italy; alice.marzimanfroni@studio.unibo.it (A.M.M.); giovanni.badiali@unibo.it (G.B.); achille.tarsitano2@unibo.it (A.T.); 2 Cellular Signalling Laboratory, Anatomy Centre, Department of Biomedical and Neuromotor Sciences (DIBINEM), University of Bologna, 40126 Bologna, Italy; mariavittoria.marvi2@unibo.it (M.V.M.); simone.lodi8@unibo.it (S.L.); giulio.vara2@unibo.it (G.V.); alessandra.ruggeri@unibo.it (A.R.); lucia.manzoli@unibo.it (L.M.); 3 Laboratoire d’Anatomie, Biomécanique et Simulation, UFR Medicine and Pharmacy, Bat D1-Porte J 6, Milétrie Street, TSA 51115, CEDEX 9, 86073 Poitiers, France; cyril.breque@simedys.com; 4 Department of Biomedical and Neuromotor Sciences (DIBINEM), University of Bologna, 40126 Bologna, Italy

**Keywords:** anatomical, reconstruction, galeo-pericranial flap, surgical simulation, cadaveric dissections, donated body, pericranium

## Abstract

Oral cavity defects occur after resection of lesions limited to the mucosa, alveolar gum, or minimally affecting the bone. Aiming at esthetical and functional improvements of intraoral reconstruction, the possibility of harvesting a new galeo-pericranial free flap was explored. The objective of this study was to assess the technical feasibility of flap harvesting through anatomical dissections and surgical procedure simulations. Ten head and neck specimens were dissected to simulate the surgical technique and evaluate the vascular calibers of temporal and cervical vessels. The procedure was therefore reproduced on a revascularized and ventilated donor cadaver. Anatomical dissections demonstrated that the mean cervical vascular calibers are compatible with superficial temporal ones, proving to be adequate for anastomosis. Perforating branches of the superficial temporal vascularization nourishing the pericranium were identified in all specimens. In conclusion, blood flow presence was recorded after anastomosing superficial temporal and facial vessels in the revascularized donor cadaver, demonstrating both this procedure’s technical feasibility and the potential revascularization of the flap and therefore encouraging its potential in vivo application.

## 1. Introduction

Head and neck defects of congenital, traumatic, infective, or neoplastic origin generally require surgical reconstruction. Resection of lesions of this district can cause significant bone, soft tissue, and skin loss. This may result in morphologic complications and functional impairment, including speech and swallowing deficits [1].

In the past, locoregional flaps were used to attempt mechanical restoration of complex defects, also obtaining esthetic refinements. The use of microvascular tissue transfer has shown higher success rates, better functional outcomes, and improved esthetics [2,3].

Although advances in radiotherapy and chemotherapy for oral squamous cell carcinoma (OSCC) have been remarkable, resection followed by reconstructive surgery is nowadays the mainstay of treatment [4]. Free tissue transfer has become an important method for surgical site restoration after primary tumor resection; it currently represents one of the most popular and reliable techniques for oral reconstructive surgery. Among these procedures, microsurgical tissue transfer has been considered the standard method for restoring postoperative functions and morphology [5,6].

The osteogenic potential of periosteal tissues has great relevance regarding the purposes of reconstruction. Among periosteal flaps, radial forearm and medial femoral condyle flaps are the most used for intraoral reconstruction [7,8,9]. However, harvesting flaps from these sites is challenging due to the small periosteal area and significant donor site morbidity. Accordingly, a search for improvement is needed; the possibility of harvesting a flap from the closer temporal region was explored. Indeed, temporal flaps are proven to be functionally reliable in the reconstruction of head and neck defects and currently part of the surgical standard for intraoral reconstruction [10].

A previous study found that the galeo-pericranial flap, in its pedicled design, is morphologically and functionally reliable for the reconstruction of defects that affect the oral cavity. In the study, eight (*n* = 8) patients underwent reconstructive surgery with this pedicled flap, nourished by superficial temporal vessels and rotated to cover defects of the upper alveolar gum or oral mucosa, all with good outcomes [11]. Even though there is limited literature describing the clinical use of this galeo-pericranial free tissue transfer, both periosteal flaps and flaps harvested from the temporal region are of common use in head and neck surgery [12,13,14,15].

This flap includes two layers, fascia and periosteum, harvested from the temporal region and accordingly supplied by the superficial temporal vascular network. It has already been described as a pedicled flap for the reconstruction of the hard palate, soft tissue, and maxillary alveolar gum [11]. However, it has not yet been used as free flap.

The main purpose of this study is to evaluate the viability of the application of a new galeo-pericranial free flap, aiming at the improvement of intraoral reconstruction of partial maxillary and mandibular defects, limited to the alveolar gum or minimally affecting the bone, which generally occur after resection of neoplastic lesions of the oral cavity.

This microvascular free flap design would require an anastomosis between superficial temporal and cervical vessels, which was simulated in this preclinical study to evaluate its potential in vivo reproduction.

## 2. Materials and Methods

It was decided to undertake an anatomical study of a galeo-pericranial free flap, articulated on cadaveric dissections. This study was divided into two main steps:

1. Vascular calibers measurement and comparison to plan an anastomosis between superficial temporal and cervical vessels;
2. Cadaveric surgical procedure simulation on ten (*n* = 10) head and neck specimens and one (*n* = 1) full-body revascularized and ventilated specimen (SimLife^®^ technology, Poitiers, France) [16].

This is a pilot prospective study of a non-pharmacological, surgical-interventional nature. It is identified by the Local Ethics Committee (CE-AVEC) as n. 173/2022/Sper/AOUBo (Evaluated on 18 May 2022).

### 2.1. Head and Neck Specimens Preservation and Staining Procedure

The analysis of the anatomical region of interest was the first step of this study. Dissections on ten (*n* = 10) donor cadavers bilaterally were performed at the Centre of Clinical Surgical Experimental and Molecular Anatomy, Department of Biomedical and Neuromotor Sciences (DIBINEM) of the University of Bologna, in the “Giovanni Mazzotti” dissecting room. The cadavers were obtained from the Body Donation Program of the University of Bologna. Cadaver age, race, height, parity, and cause of death were recorded. A technical-grade embalming solution was used (PanReac Applichem^®^, Barcelona, Spain). The study was conducted in accordance with the Code of Ethics of the World Medical Association (Declaration of Helsinki). Anatomical dissections included ten (*n* = 10) head and neck fresh specimens, three (*n* = 3) of which underwent a plastination procedure with a colored latex solution, on both sides of each head. The donors were two (*n* = 2) males and eight females (*n* = 8), all Caucasian, ranging from 58 to 91 years of age, as shown in Table 1.

The anatomical head and neck fresh preparations were kept at a temperature between −18 and −20 °C, with the purpose of obtaining optimal preservation. During the study, it was necessary to unfreeze the specimens between 24 to 48 h before the dissection to have an adequate texture of the tissues.

With the intent to evaluate the vascular supply of the temporal and cervical regions, colored latex solutions were injected into the vessels of three out of ten (3/10) donors. The carotid artery and jugular vein were isolated from the surrounding tissues and cannulated with a 0.5 mm curved blunt steel needle inserted in the vessel for 2 or 3 cm approximately. The artery was filled with red latex stain while the vein was filled with blue latex stain. After injection, the structures were secured with 6–0 silk sutures to prevent leakage of the stain and dislocation.

This procedure was fundamental for the identification of the superficial temporal, facial, and superior thyroid vessels and for the study of the micro-vascularization, including the perforating vessels nourishing the pericranium.

### 2.2. Preparation of the Revascularized and Ventilated Body

The body of one Caucasian male (aged 76) was donated to the Center of Clinical Surgical Experimental and Molecular Anatomy and kept at a temperature between −18 and −20 °C, with the purpose of obtaining optimal preservation (Table 2). The body was progressively thawed (at 16 °C) over three days, on the occasion of the day dedicated to simulation training in healthcare with a realistic environment organized by our Center in collaboration with Simedys^®^ company [16].

The day before SimLife^®^ simulation session, the body was prepared in the perspective of a surgical simulation scenario with a hemodynamic and respiratory control device. In detail, some cannulas were placed by bilateral incisions (cervical, femoral, and brachial), at both arterial (input) and venous (output) level, as previously described [17]. Ventilation was provided through tracheotomy (30 Fr), and stomach emptying was obtained via a nasogastric tube (21 Fr, 120 cm). The native blood was then washed with water at low pressure (0.8 bar) and at a temperature of 30 °C. Before the start of the simulation and during the whole procedure, the physiological characteristics of the subject (heart and respiratory rate and blood pressure) were computer monitored and adapted as needed. SimLife^®^ technique guaranteed re-vascularized, re-colored, and warmed inner organs through specific mimicking-blood liquid [18].

### 2.3. Vascular Calibers Measurement

During dissection of each head and neck specimen, superficial temporal and cervical vascular calibers were measured using a sterile disposable ruler, and the course of each vessel was recorded to identify anatomical variants.

After a coronal incision, the superficial temporal artery (STA) and superficial temporal vein (STV) were identified in each pre-auricular region. The skin and subcutaneous tissue were dissected to separate the vessels from the surrounding tissues and measure them before flap harvesting.

The calibers of STA and STV were recorded before their bifurcation into frontal and parietal branches.

The recipient-site vessels were identified after a cervical incision, followed by careful dissection of the superficial layers. The facial artery and vein were measured in their course at the level of the mandible, superficially, while superior thyroid vessels were isolated and evaluated in their path along the lateral border of the thyrohyoid muscle.

### 2.4. Surgical Procedure Simulation

After vascular study, the simulation of free flap harvesting for the reconstruction of mandibular defects was performed on ten (*n* = 10) head and neck specimens to evaluate the applicability of the surgical procedure, as shown in Video S1 (Supplementary Materials).

#### 2.4.1. Identification of the Superficial Temporal Vascular Network

A coronal incision using a number 15 scalpel blade was the first step toward the identification of the superficial temporal vessels and their branches.

After the incision, the frontal and parietal branches of the STA and STV were identified, and their course was followed to find the main vessels in the preauricular region.

Once the subcutaneous tissue was dissected anteriorly and inferiorly for several centimeters, it was possible to evert the cutaneous flap so that the tissues underneath could be exposed. This procedure helped to preserve the vessels lying in the deeper layer; the position of the superficial temporal vein, which is generally found superficial to the artery, dictated the depth of dissection.

Depending upon the operative procedure, it was decided to perform flap harvesting on the parietal branches of the superficial temporal vessels with the intent to avoid any surgical complication due to injury to the temporal branch of the facial nerve, which runs along the path of the frontal branches of the STA and STV.

#### 2.4.2. Flap Harvesting

Proceeding in a cranial-caudal direction to harvest the flap, the path of the vessel layer was followed until reaching the temporal fascia, which was included in the flap.

To complete the harvesting procedure, the pericranium was peripherally incised once the area of relevance had been marked. Periosteal elevators were used to lift the pericranial layer of the flap and continue the dissection, taking great care to avoid tearing the tissue. During flap elevation, it was possible to notice the presence and the course of perforating vessels nourishing the pericranium.

Dissection proceeded in the sub-pericranial layer until the superior temporal line was reached. This anatomical landmark indicated the fascial insertion into temporal bone and the spreading of the pericranial layer.

Dissection proceeded in the superficial plane to the deep temporal fascia (temporoparietal fascia), which remained attached to the temporal muscle. Instead, part of the superficial fascia covering the temporal muscle was included in the flap. At this point, the anterior helix was reached, and the flap could be elevated, isolating the main vessels in the preauricular region. This allowed to complete the flap harvesting, mobilizing the deep portion of the galeo-pericranial tissue, and finally detaching the flap.

Concerning flap harvesting, eighteen (*n* = 18) galeo-pericranial flaps were elevated (nine specimens bilaterally), since in one of the donors the superficial temporal network was not possible to isolate (Specimen D).

#### 2.4.3. Preparation of the Recipient Site 

After flap harvesting, the study focused on the cervical region, to find the vessels designated for the anastomosis. Firstly, the facial and superior thyroid vessels were isolated in their course, carefully separating them from the surrounding tissues.

Once the vessels were identified and measured, an intraoral vestibular approach was undertaken to reach the mandible and simulate the surgical defect. The current study was carried out adopting the following inclusion criteria: all defects included the alveolar gingiva or minimally affected the bone, with an overall maximum diameter that did not exceed 3 cm.

The lower lip was everted and an incision in the oral mucosa was placed at the level of the canine, premolar or molar regions. A scalpel blade n. 15 was oriented perpendicular to the bone when dissecting above the mental foramen, to prevent incision of the mental nerve. The mentalis muscle was also incised until the bone was encountered and a periosteal elevator was used to strip the muscle. At this point, the mandible was completely freed from the mucosal and muscular tissues covering it and it was possible to perform an osteoplasty or, in some cases, a superficial marginectomy using Mectron Piezosurgery^®^ device (Mectron, Loreto, Italy).

#### 2.4.4. Insetting of the Flap

Once the demolition was completed, it was possible to plan the reconstructive procedure.

The first step was the insetting of the flap on the recipient site prior to beginning the anastomosis. The fascial-periosteal tissue was plied and positioned to fully cover the defect and fixed it to the surrounding structures. A surgical drill was used to make two holes in the bone at both sides of the defect to secure the flap to the mandible. A total of 6–0 silk sutures were also placed on the intraoral periosteum, on the adjacent oral mucosa, on the anterior portion of the masseter muscle, and on the posterior portion of the mental muscle.

#### 2.4.5. Vascular Anastomosis

After insetting, the vascular calibers of the facial and superior thyroid vessels were compared to the superficial temporal ones to decide the most appropriate in terms of diameter for each specimen. The anastomosis was thus performed with 8-0 polypropylene sutures, using the Zeiss OPMI MDO S5^®^ surgical microscope (Carl Zeiss International, Oberkochen, Germany). The superficial temporal artery was anastomosed with the facial artery in its superficial course, at the level of the mandible, in two of the colored specimens bilaterally. The superior thyroid vessels were not used as they seem to have thinner walls in cadaveric preparations, especially after latex staining.

#### 2.4.6. Surgical Simulation on a Revascularized and Ventilated Cadaver

After completing the dissection of ten (*n* = 10) head and neck specimens bilaterally, surgical procedure was lastly simulated on a revascularized and ventilated donor cadaver, as shown in Video S1 (Supplementary Materials). SimLife^®^ technology is designed to offer realistic scenarios and experiences to medical and surgical participants by facilitating the reventilation and revascularization of bodies donated to science.

The constant blood flow provided by this innovative technology guarantees a more realistic vascular anastomosis simulation.

In this last specimen, superficial temporal and facial vessels were anastomosed thanks to the use of the Leica^®^ M320 F12 surgical microscope (Leica Microsystems, Wetzlar, Germany): this allowed a more precise evaluation of their compatibility in terms of diameter as no blood leakage was observed after finalizing the anastomosis. Once the surgical approach was completed, Siemens Acuson Redwood^®^ doppler ultrasound was used to analyze the presence and entity of blood flow within the neoanastomosis.

## 3. Results

### 3.1. Vascular Calibers Evaluation

**Figure 1:**
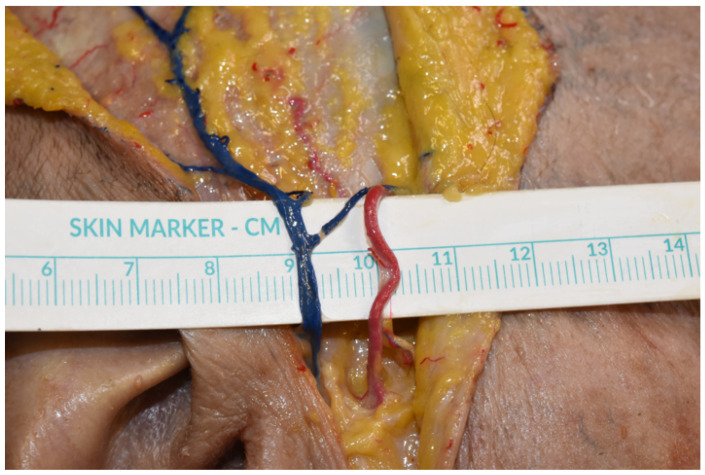
Superficial temporal vein and artery in preauricular region. The figure shows the superficial temporal vein (blue stain) and artery (red stain) which were isolated and measured in the preauricular region after lifting the cutaneous flap.

Among the many factors that influence surgical planning, the selection of appropriate recipient vessels is essential to obtain flap success. During dissection of each head and neck specimen, the vascular calibers of superficial temporal (Figure 1), facial, and superior thyroid arteries and veins were measured bilaterally.

Each diameter of the isolated vessels was recorded, particularly focusing on the arteries, as shown in Table 3.

The results of the measurements showed the following mean vascular calibers, with the associated error (standard deviation):

- 2.17 ± 0.44 mm for the superficial temporal arteries;
- 2.53 ± 0.53 mm for the facial arteries;
- 1.95 ± 0.51 mm for the superior thyroid arteries;
- 1.7 ± 0.5 mm for the superficial temporal veins;
- 1.93 ± 0.58 mm for the facial veins;
- 1.67 ± 0.71 mm for the superior thyroid veins.

Analyzing the data of the cadaveric study, no significant discrepancy in the mean vascular calibers of interest was recorded:

- The STA was 0.36 mm smaller in diameter when compared to the facial artery and 0.2 mm larger than the superior thyroid artery;
- The STV was 0.23 mm smaller in diameter than the facial vein and 0.03 mm larger than the superior thyroid vein.

### 3.2. Vascular Course Evaluation

The course of the three previously mentioned vascular networks was followed and the anatomical variants were recorded as shown in Table 3.

It was possible to notice that the superior thyroid arteries (3, 6) were more subject to variability in the specimens dissected. It was also possible to observe how the superficial temporal arteries (1, 4) in their parietal branches could divide and form unusual anastomoses with the occipital pedicle, none with real clinical significance for what concerns the blood supply to the pericranium. No significant variant was observed when dissecting the facial arteries. In particular, the following variations were recorded:

- Absence of a common trunk between right superior thyroid artery and right superior laryngeal artery in two of the donors;
- Left superior thyroid artery directly originating from the left common carotid artery in one of the donors;
- Left superior thyroid artery originating from the carotid bulb in one of the donors;
- Absence of a common trunk between left superior thyroid artery and superior laryngeal artery in one of the donors.

Nonetheless, the unusual courses of the isolated vascular structures did not appear to have any clinical relevance in terms of flap harvesting and anastomosis.

### 3.3. Surgical Procedure Simulation on Ten Donor Cadavers

**Figure 2:**
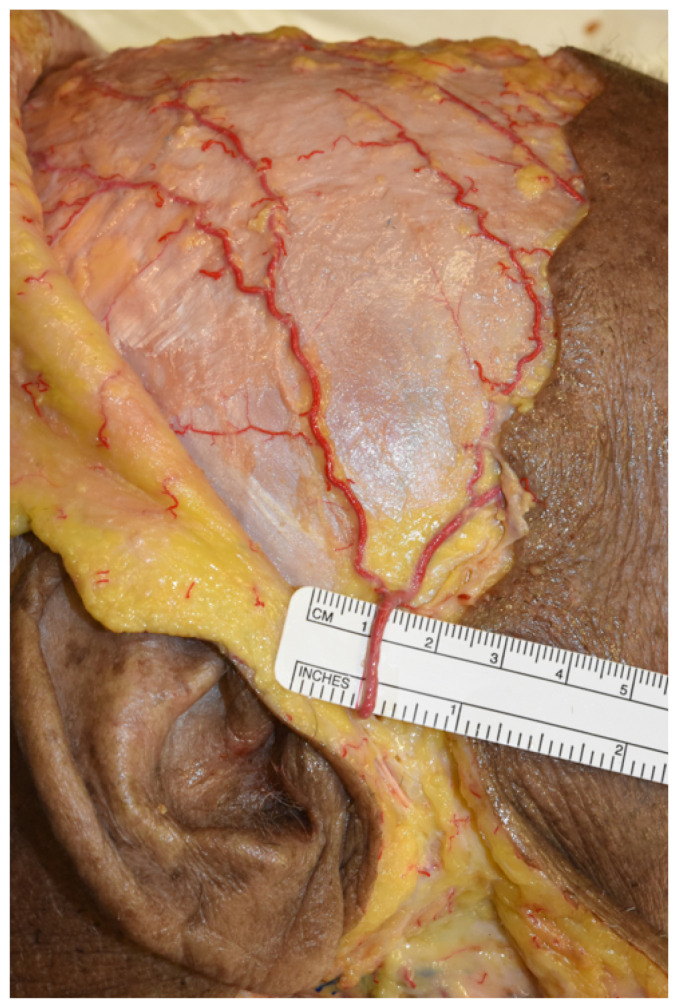
Exposure of the superficial temporal artery and its branches. The figure shows the exposure of the superficial temporal artery and its frontal and parietal branches after coronal incision.

The simulation of free flap harvesting for the reconstruction of mandibular defects was performed. A coronal surgical approach was the first choice due to its many advantages; it provides excellent access to the area of interest with minimal complications and most of the surgical scar is hidden within the hairline, even when the incision extends to the preauricular region at the level of the helix [19,20] (Figure 2).

**Figure 3:**
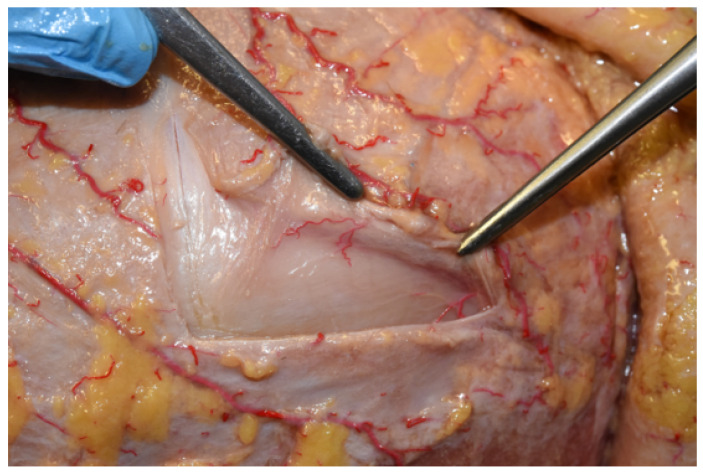
Perforating vessels to the pericranium. The figure shows perforating branches of the superficial temporal artery observed during dissection, nourishing both the galeal and pericranial layer.

During dissection, perforating vessels arising from the superficial temporal network and nourishing the pericranium were identified in each specimen. These vessels seemed to pass through the galea aponeurotica until they reached the deeper pericranial layer. As a matter of fact, these are fundamental for the vitality of the flap; injuring these vessels could compromise the engraftment of the flap to the recipient site (Figure 3).

**Figure 4:**
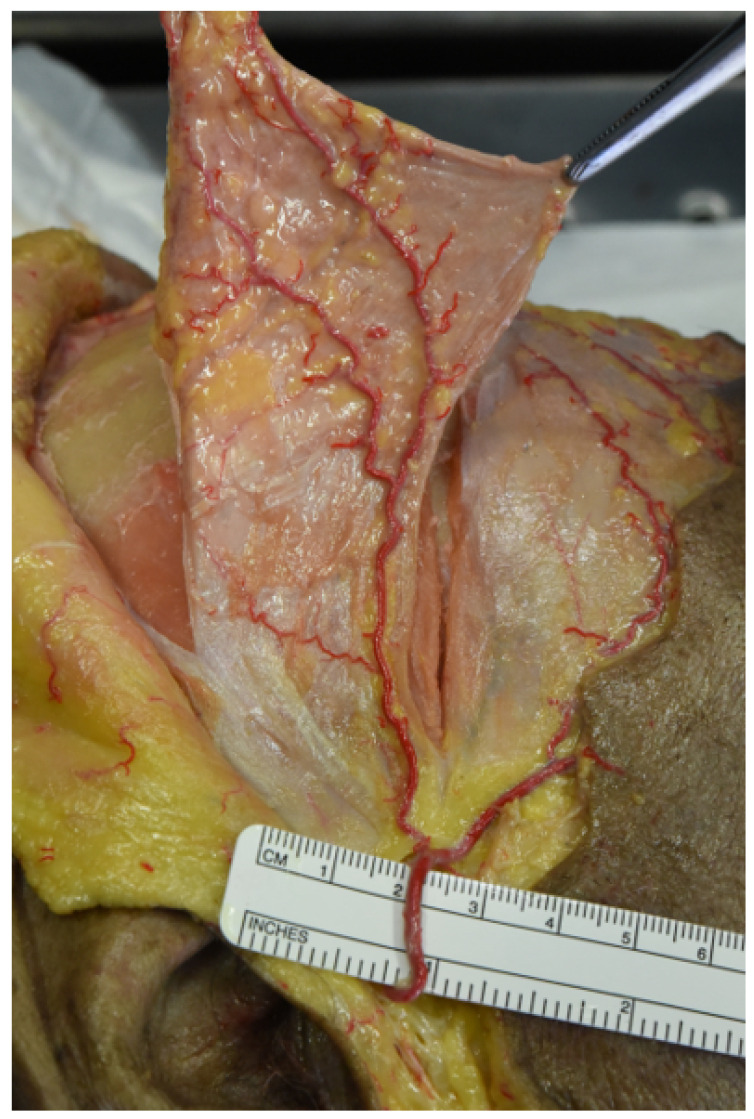
Elevation of the flap pedicled on the parietal branch of the superficial temporal artery (STA). The figure shows the elevation of the galeo-pericranial flap, including part of the superficial temporal fascia, pedicled on the parietal branch of the superficial temporal vessels.

At this point, galeo-pericranial flaps were elevated and detached from the temporal region (Figure 4).

The maximum width of the flaps with superficial temporal fascia included was 15 cm, the extension of the flap depended on the vascular pattern of the superficial temporal network for each donor.

The average galeo-pericranial layer of these flaps ranged between 6 and 8 cm × 9 and 13 cm, which was sufficient to cover the mandibular defects reproduced.

Once the flaps were positioned on the recipient site, an end-to-end anastomosis between superficial temporal and cervical vessels was performed. This type of anastomosis is generally preferred because of its flow characteristics and technical facility. However, it should not be performed if there is a greater than 50 percent discrepancy in diameter between the two vessels to be joined.

This incongruity was not recorded in any of the vessels used for the anastomosis in the simulations. Arterial anastomoses were performed in seven (*n* = 7) head and neck specimens bilaterally; facial artery was the preferred recipient vessel due to its larger caliber in the specimens dissected and close proximity to the recipient site. The reduced thickness caused by the emptiness of venous vessels in the donor cadavers limited the possibility to reproduce a realistic venous anastomosis in these head and neck specimens.

### 3.4. Surgical Procedure Simulation on a Revascularized and Ventilated Specimen

After the evaluation of the applicability of this surgical technique on ten (*n* = 10) donor cadavers, the procedure was undertaken on one (*n* = 1) revascularized and ventilated specimen. The addition of blood-mimicking fluid supply in this donor cadaver guaranteed a more realistic simulation, especially regarding the vascular anastomosis between the superficial temporal and facial vessels.

**Figure 5:**
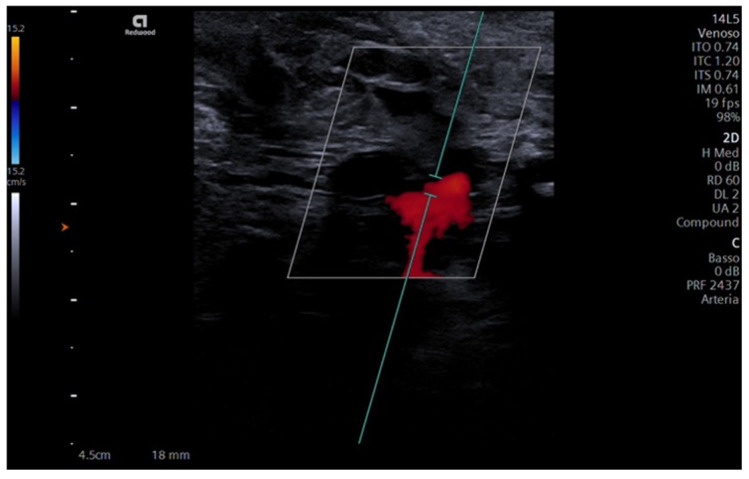
Doppler ultrasound. The figure shows the Color-Doppler ultrasound evidencing blood-mimicking fluid presence in the anastomosis between superficial temporal and facial arteries.

Furthermore, blood flow presence and entity in the neoanastomosis were recorded using doppler ultrasound, with favorable outcomes (Figure 5).

Surgical procedure simulation resulted in the technical feasibility of galeo-pericranial flap harvesting, insetting on the mandibular defect, and anastomosis between superficial temporal and facial vessels.

## 4. Discussion

Surgical defects of neoplastic, post-irradiation, or infective nature that affect the oral cavity generally require reconstruction. Without restoration of the surgical site, patients would incur morphological complications and functional impairment, with difficulties in swallowing and speech [1]. Local flaps seem to be the best option for the reconstruction of small and medium defects, while for larger lesions, microvascular tissue transfer has shown better functional and esthetic outcomes [2,3,5,6].

Periosteum composite flaps have demonstrated great relevance regarding the purposes of reconstruction of defects that affect both the intraoral mucosa and bone. Among these flaps, radial forearm and medial femoral condyle flaps are the most used. However, harvesting flaps from these sites is challenging due to the small periosteal area and significant donor site morbidity [7,8,9]. For these reasons, the possibility of harvesting a periosteal flap from the closer temporal region was explored. These flaps are proven to be functionally and esthetically reliable for intraoral reconstruction after surgical ablative procedures [10]. The temporal muscle myofascial flap is a versatile option for surgery of moderate- to large-sized maxillofacial defects; although scarring at the site of incision is insignificant and alopecia is generally never observed, hollowing in the temporal region could be a complication of the transposition of this flap [21,22].

Galeo-pericranial pedicled flaps demonstrated improved functional and morphological outcomes, together with good intraoral healing and remucosization at the recipient site, no permanent complications, and minimum donor site morbidity when used for maxillary oral mucosa reconstruction. Given the promising results of this previous clinical study, it was decided to continue the investigation by analyzing the possible applications of the galeo-pericranial flap as free microvascular tissue, which would allow periosteal transfer for reconstruction of surgical defects that could not be reached by the pedicled flap. For instance, defects that affect the mandibular region or anterior floor of the mouth are far from the arc of rotation of the superficial temporal vascular pedicle; patients with neoplastic lesions, osteitis, or osteoradionecrosis of the lower jaw area could therefore benefit from this free galeo-pericranial transfer.

To overcome this problem, ultrathin temporoparietal fascia flaps (TPFF) and temporoparietal galea flaps (TPGF) can be used to reconstruct a wide variety of defects in the craniofacial area, including all types of intraoral and even mandibular reconstructions [21,23]. Given the minimal donor site morbidity and absence of functional or esthetic sequelae of this technique, the possibility of including a periosteal layer in these fibromuscular sheets was explored.

Although galeal flaps have not yet been described in literature in their microvascular free design, their use as pedicled flaps appears to be a versatile option for the reconstruction of small to moderate defects of the head and neck region [11]. A previous clinical study [11] assessed the application of a galeo-pericranial flap in its pedicled form in reconstructive surgery for defects affecting the maxillary region; all patients showed good healing, with the surgical defects being fully covered and restored. No major vascular complications and no necrosis nor dehiscence were noted, no functional nor esthetic issues at either the recipient or donor site were encountered. In addition, most of the coronal incision was hidden within the hairline and no noticeable scar was left. The promising results of this clinical study encouraged further assessments regarding the viability of application of a galeo-pericranial free microvascular flap.

This anatomical study revealed that the superficial temporal network provides a consistent supply to the temporal region; studying the course of the STA, perforating vessels running from the overlying galea deep into the pericranium were found, nourishing both layers.

The superficial temporal vessels are readily available, and their suitability for microvascular tissue transfer in head and neck reconstruction has been reported by numerous authors [24,25,26,27].

The superficial temporal vein has a complex pattern with numerous branches and connections, which make it a safe option for dissection and anastomosis even if the ipsilateral internal jugular vein is ligated [25].

For their reliability in the microvascular reconstruction of the upper two-thirds of the face, superficial temporal vessels have been recommended by numerous authors as a first option for recipient vessels in oncological midface reconstruction [25,26,28,29]. However, few publications report the superficial temporal network as donor site vessels, although the results seem satisfactory [30,31,32,33].

The cervical region has an extensive bilateral vascular network, which is readily accessible for free flap transfers. Facial and superior thyroid vessels are first choice recipient vessels for microvascular head and neck reconstruction of middle- and lower-third defects [25,29,34]. These vessels are generally easy to identify and dissect, except for some cases such as patients with a history of previous surgery and/or radiotherapy. Anyhow, the use of surgical microscopes could lower the risk of damage to facial and superior thyroid vessels and increase the success rate of anastomosis in previously treated necks [29].

The data collected in this study are comparable to other anatomical cadaveric studies in the literature, which have identified an average diameter of the superficial temporal artery from 1.8 to 2.7 mm in the preauricular region [24]. In agreement with the current study, the facial arteries seemed to have a slightly larger diameter, with values ranging from 2.3 to 2.6 mm at the lower edge of the mandible, while there is no clear anatomical evaluation regarding the caliber of cadaveric superior thyroid vessels in the literature.

However, in vivo studies showed that these vascular calibers are not always similar to the ones measured in cadavers [24]. Indeed, it should be considered that vascular walls tend to modify their consistency, their elasticity, and consequently, their size postmortem. Commonly, the calibers of the superficial temporal arteries in vivo range between 1.8 and 2.2 mm while the veins range from 2.0 to 3.0 mm. The diameter of the facial artery ranges from 2.0 to 2.8 mm, whereas the caliber of the vein might be found between 2.2 and 3.2 mm [35]. This shows how venous walls tend to retract when empty, being therefore smaller in cadavers. Nonetheless, the results of these in vivo CT studies demonstrated that these vascular calibers are generally similar within the same patient, indicating that vessel size is not a limitation [24].

It should however be considered that three of the donor cadavers’ vessels were injected with latex solution which, in some cases, could increase vessel calibers, especially venous ones. This could be considered a limitation as stained vessels might be less reliable for study purposes.

A positive confirmation was given by the simulation of the surgical procedure through the use of SimLife^®^ technology, which denoted the presence of blood flow in the vascular anastomosis between superficial temporal and facial vessels and the consequent nourishment to the galeal and pericranial layers of this flap.

Ultrasound with Color-Doppler can be useful to evaluate the success of the procedure [36]; however, micro-bubbles inherently present in the artificial blood generate minor artifacts, as shown in Figure 5, and further studies are needed to optimize the scanning settings to improve the quantitative evaluation in this context.

This dissection was additionally essential for the identification, measurement, and anastomosis of venous vessels, which appeared smaller in non-vascularized preparations as their walls seemed to retract when empty.

It is important to underline that surgical simulations on donor cadavers, however realistic, present some limitations regarding the different consistency of the tissues, the absence of the physiological pulsatility of blood flow, and the impossibility of carrying out post-operative evaluations.

The overall results of these anatomical studies have encouraged us to evaluate the applicability of the galeo-pericranial flap in vivo.

Superficial temporal, facial and superior thyroid vessels are proven to be adequate for anastomosis in flap harvesting for head and neck surgical defects reconstruction [25,26,28,29].

## 5. Conclusions

This anatomical study demonstrated the technical feasibility of the free flap harvesting in surgical procedure simulations on donor cadavers. The presence of blood flow was recorded after anastomosing superficial temporal and facial vessels in a vascularized donor cadaver using SimLife^®^ technology, testifying the potential flap revascularization nourishing both the galeal and pericranial layers.

Despite the limitations represented by the different venous consistency and the absence of physiological pulsatile blood flow, the compatibility of superficial temporal, facial, and superior thyroid vessels in terms of mean caliber in cadaveric dissections and the successful outcomes of surgical procedure simulation on vascularized specimens further strengthens the hypothesis of potential success of an anastomosis between these vessels in vivo.

The galeo-pericranial free flap could represent a valid option for functional and esthetic improvement of intraoral reconstruction of partial maxillary and mandibular oncological defects, limited to the alveolar gum or minimally affecting the bone.

## Figures and Tables

**Table 1:** Demographic characteristics of the donors and staining preparation.

	Sex	Age	Ethnicity	Stain
SPECIMEN A	M	88	Caucasian	/
SPECIMEN B	F	58	Caucasian	/
SPECIMEN C	F	60	Caucasian	/
SPECIMEN D	F	63	Caucasian	/
SPECIMEN E	F	91	Caucasian	/
SPECIMEN F	F	83	Caucasian	/
SPECIMEN G	F	73	Caucasian	/
SPECIMEN H	M	75	Caucasian	Latex solution
SPECIMEN I	F	69	Caucasian	Latex solution
SPECIMEN J	F	67	Caucasian	Latex solution

**Table 2:** Demographic characteristics of donor K (revascularized and ventilated body) and staining preparation.

	Sex	Age	Ethnicity	Stain
SPECIMEN K	M	76	Caucasian	Blood-mimicking fluid

**Table 3:** Arterial calibers and anatomical variants.

	Right			Variants	Left			Variants
	Superficial Temporal (1)	Facial (2)	Superior Thyroid (3)		Superficial Temporal (4)	Facial (5)	Superior Thyroid (6)	
SPECIMEN A	3 mm	3 mm	2 mm	3	3 mm	3 mm	2.5 mm	4
SPECIMEN B	3 mm	3 mm	2 mm		2 mm	2 mm	2 mm	6
SPECIMEN C	2 mm	ND	2 mm		2 mm	ND	ND	
SPECIMEN D	ND	3.5 mm	2.5 mm		ND	3.5 mm	2.5 mm	
SPECIMEN E	2 mm	2.5 mm	2 mm	1	2 mm	2.5 mm	3 mm	4
SPECIMEN F	1.5 mm	3 mm	1.5 mm		1.5 mm	2 mm	2 mm	6
SPECIMEN G	2 mm	2 mm	2 mm	3	2 mm	1.5 mm	2 mm	6
SPECIMEN H	2.5 mm	2.5 mm	2 mm		2.5 mm	2 mm	ND	
SPECIMEN I	2 mm	2 mm	1 mm	1	2 mm	2.5 mm	1.5 mm	
SPECIMEN J	2 mm	ND	1.5 mm		2 mm	ND	1 mm	

## Data Availability

All data are available upon request to https://site.unibo.it/centro-anatomico/it.

## Supplementary Materials

The following supporting information can be downloaded at: https://www.mdpi.com/article/10.3390/jcm12247533/s1. Video S1: surgical procedure simulation on a revascularized and ventilated donor cadaver. This video shows the surgical procedure simulation on a revascularized and ventilated donor cadaver with the aid of SimLife^®^ technology. An emicoronal incision was the first step toward the identification of the superficial temporal vessels and their branches in the preauricular region. The path of the vessel layer was followed until reaching the temporal fascia, which was included in the flap (surgical step 1). In this case, it was decided to perform flap harvesting on the frontal branch of the superficial temporal vessels. To complete the harvesting procedure, the pericranium was peripherally incised and lifted using periosteal elevators. Dissection continued in the plane superficial to temporoparietal fascia, which remained attached to the temporal muscle, while part of the superficial fascia was included in the flap (surgical step 2). At this point, the flap could be elevated, mobilizing the deep portion of the galeo-pericranial tissue, and finally detached (surgical step 3). After flap harvesting, the dissection focused on the cervical region, to find the most appropriate vessels for the anastomosis. In this case, the facial vessels were isolated in their course, carefully separating them from the surrounding tissues (surgical step 4) and anastomosed with superficial temporal vessels thanks to the use of the Leica^®^ M320 F12 surgical microscope (surgical step 5). No blood-mimicking fluid leakage was observed after finalizing the anastomosis and flow presence and entity were recorded using Doppler ultrasound, with favorable outcomes (surgical step 6).
