# Supplementary material for: Anatomical Study of the Application of a Galeo-Pericranial Flap in Oral Cavity Defects Reconstruction
Source: J Clin Med. 2023 Dec 6;12(24):7533. doi: 10.3390/jcm12247533 (PMC10743416; doi:10.3390/jcm12247533)
Supplement: Supplementary file 1 [file jcm-12-07533-s001.zip › jcm-2745795-supplementary.pdf]

Supplementary Materials:

Video S1: This video shows the surgical procedure simulation on a revascularized and ventilated donor cadaver with the aid of SimLife® technology. An emicoronal incision was the first step toward the identification of the superficial temporal vessels and their branches in the preauricular region. The path of the vessel layer was followed until reaching the temporal fascia, which was included in the flap. (Surgical step 1)

In this case it was decided to perform flap harvesting on the frontal branch of the superficial temporal vessels. To complete the harvesting procedure, the pericranium was peripherally incised and lifted using periosteal elevators. Dissection continued in the plane superficial to temporoparietal fascia, which remained attached to the temporal muscle, while part of the superficial fascia was included in the flap. (Surgical step 2)

At this point, the flap could be elevated, mobilizing the deep portion of the galeo-pericranial tissue, and finally detached. (Surgical step 3)

After flap harvesting, the dissection focused on the cervical region, to find the most appropriate vessels for the anastomosis. In this case, the facial vessels were isolated in their course, carefully separating them from the surrounding tissues (Surgical step 4) and anastomosed with superficial temporal vessels thanks to the use of the Leica® M320 F12 surgical microscope. (Surgical step 5)

No blood-mimicking fluid leakage was observed after finalizing the anastomosis and flow presence and entity were recorded using Doppler ultrasound, with favorable outcomes. (Surgical step 6)

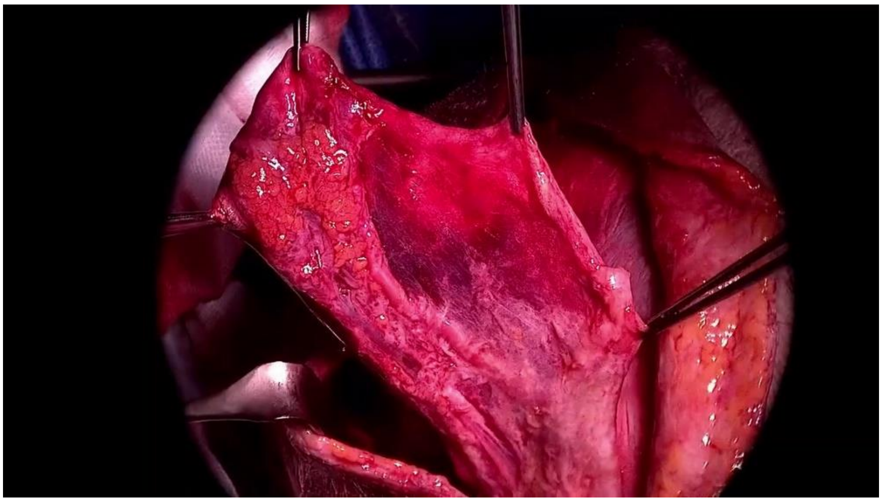

Still picture of Video S1.
